# Fundamental Limits on Wavelength, Efficiency and Yield of the Charge Separation Triad

**DOI:** 10.1371/journal.pone.0036065

**Published:** 2012-06-01

**Authors:** Alexander Punnoose, Liza McConnell, Wei Liu, Andrew C. Mutter, Ronald Koder

**Affiliations:** 1 Instituto de Física Teórica, Universidade Estadual Paulista, São Paulo, Brazil; 2 Department of Physics, City College of the City University of New York, New York, New York, United States of America; University of Illinois, United States of America

## Abstract

In an attempt to optimize a high yield, high efficiency artificial photosynthetic protein we have discovered unique energy and spatial architecture limits which apply to all light-activated photosynthetic systems. We have generated an analytical solution for the time behavior of the core three cofactor charge separation element in photosynthesis, the photosynthetic cofactor triad, and explored the functional consequences of its makeup including its architecture, the reduction potentials of its components, and the absorption energy of the light absorbing primary-donor cofactor. Our primary findings are two: First, that a high efficiency, high yield triad will have an absorption frequency more than twice the reorganization energy of the first electron transfer, and second, that the relative distance of the acceptor and the donor from the primary-donor plays an important role in determining the yields, with the highest efficiency, highest yield architecture having the light absorbing cofactor closest to the acceptor. Surprisingly, despite the increased complexity found in natural solar energy conversion proteins, we find that the construction of this central triad in natural systems matches these predictions. Our analysis thus not only suggests explanations for some aspects of the makeup of natural photosynthetic systems, it also provides specific design criteria necessary to create high efficiency, high yield artificial protein-based triads.

## Introduction

Solar energy conversion machines found in nature utilize a number of small molecules, called cofactors, which serve as discrete sites for the binding of a single electron [1]. Charge separation in these proteins is effected via a cascade of several individual electron transfer (ET) events initiated by the absorption of a photon at a central cofactor termed the primary-donor [2]. These protein machines typically contain numerous cofactors arranged so as to enable the movement of the electron and the oxidizing equivalent away from the primary-donor in opposite directions [3], [4]. The resultant potential energy is then coupled to some chemical reaction or reactions which create a storable, diffusable form of chemical energy.

**Figure 1:**
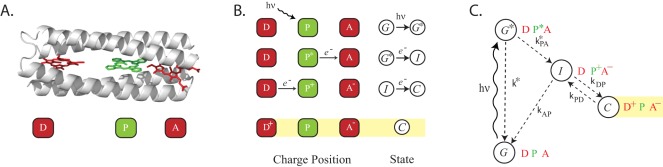
Structure and function of the photosynthetic triad. (A) Molecular detail of an idealized artificial charge separation construct, a self-assembling de novo designed protein. (B) Discrete steps in the formation of the charge separated state: The primary-donor molecule P in the ground state configuration 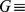 DPA absorbs a photon of the correct frequency to form 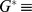 D
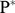
A, where 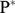 is the photoexcited state of P. The excited electron transfers to the acceptor cofactor, A, forming the intermediate state 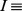 DP^+^A^−^. The donor cofactor, D, then transfers an electron into P, resulting in the charge separated state 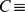 D^+^PA^−^. (C) Energy level diagram of the states in B. The *k*-variables denote the corresponding microscopic single-electron ET rates. In this scheme, the direct long range tunneling between D and A (i.e., 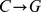) and the ‘thermal back reaction’ [33] between P and A (i.e., 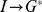) are not considered. As explained in the main text, their magnitudes can be significantly suppressed without affecting the efficiency and yield.

Chemists have made an intensive effort over the past forty years to recreate the charge separation capability of these devices in synthetic systems [5]–[11]. The minimal construct that can achieve long-lived charge separation contains a primary-donor along with two other cofactors to facilitate the separation and prevent the fast relaxation of the electron back to the groundstate of the primary-donor (see Figure 1A). This has been termed the photosynthetic cofactor triad (PCT) [7], [11]–[13]. Research efforts have aimed at engineering protein-based PCTs, either through the reengineering of natural proteins [14], [15] or de novo design of new artificial proteins [16], [17]. An optimal PCT construct will maximize the yield of the charge separated state and minimize energy loss while maintaining the state for as long as necessary before decaying to the groundstate. These performance metrics are intimately related to the microscopic ET rates which themselves are a function of the reduction potentials and the spatial arrangement of the three cofactors. Given the large expense and long time scale of these design efforts [2], [18]–[20], it is important to understand the optimal structure and properties of this molecule from the beginning of the design process. The key is to identify the set of microscopic parameters which when manipulated can effect maximum benefit during the design process.

Clearly, numerical simulations of the rate equations to map out the optimal set of ET rates for the entire construct involve a large parameter space [21]. For this reason there has been little theoretical analysis of the optimal structure and properties of the cofactor triad and its many sequential ETs. There are several semiclassical equations which predict ET rates that are well validated, in particular the semiclassical Marcus expression [22]–[26]. These are all complicated functions with a number of terms. The challenge in a complex system such as the PCT is to select the formalism which will give a meaningful analytical expression for its behavior. For example, Cho and Silby, in 1995, derived the time-dependent behavior of a molecular dyad structure composed of two cofactors and three states in the limit of a very large reduction potential difference between the excited state primary donor and the acceptor site [27].

In this work, we solve the rate equations analytically for a generic molecular triad with four states and obtain closed-form expressions relating the lifetime and yield of the charge separated state to the ET rates. The equations allow us to isolate the relevant ratios of rate constants that control the yield and the lifetime. These conditions are used to set bounds on the physical distances and potentials that makeup the PCT using a standard semiclassical model which incorporates Marcus theory for the 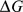 dependence and an exponential drop-off of the ET rate with distance [28] as parameterized in the Moser-Dutton ruler [29], [30]. We report two major findings: first, that the highest yield occurs when the primary-donor cofactor is closest to the acceptor cofactor and second, that the highest yield and efficiency occurs when the absorption frequency of the primary-donor is more than twice the reorganization energy of the first electron transfer. Interestingly, we demonstrate that natural systems seem to obey these rules despite their much higher degree of complexity.

## Methods

The basic PCT arrangement for long lived charge separation is depicted schematically in Figure 1A [7], and the microscopic steps leading to charge separation are shown explicitly in Figure 1B and energetically in Figure 1C: upon photoexcitation of the site of charge separation or primary-donor (P) to 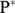, the excited electron transfers to an acceptor molecule (A). A donor molecule (D) then transfers an electron to P, thus blocking the unproductive charge recombination via back electron transfer, to create a fully charge separated state, 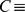 D^+^PA^−^.

The state *C* principally relaxes back to the ground state, 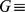 DPA, by one of two mechanisms: direct long ET between A^−^ and D^+^, or a two step recombination process via the intermediate state, 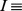 DP^+^A^−^, followed by electron transfer from A^−^ to P^+^, i.e, from state 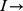 G. When the molecules are arranged linearly, as in Figure 1A, the first short-circuit reaction mechanism is considerably suppressed, and is therefore neglected in our model. The ET rates for the two step process are 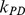 and 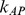, respectively. The reverse transition back to the excited state 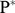 from A is also suppressed; below we demonstrate that the corresponding ET rate is exponentially suppressed for energy differences larger than 60–100 meV between 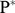 A and P^+^A^−^, which we show is much less than 10% of the output energy and therefore does not affect our general conclusions. Similarly, since the energy difference for the ET from *P* to *A* is in the eV range, thermal excitation from the groundstate to the acceptor is not considered at room temperatures.

The master equations describing the transitions between the states corresponding to the scheme in Figure 1C are:
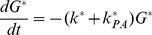
(1a)

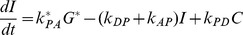
(1b)

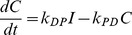
(1c)

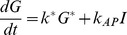
(1d)


The transition rates between different configurations is governed by the *microscopic* ET rates. The specific ET involved is encoded in the subscript, for example, 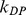, denotes the ET rate for the 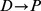 transition. The complete list of transitions are: 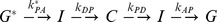 and 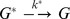. As explained earlier, the 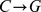 short-circuit and the reverse 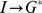 transitions are suppressed in our scheme. The rate 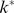 is the combined direct relaxation rate, fluorescent and otherwise, from the photoexcited state 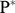 to its groundstate P.

Setting either of the two rates 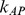 or 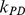 to zero in Equation 1 prevents the state *C* (the charge separated state) from decaying into the ground state *G* creating a steadystate at long times. A finite 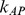 and/or 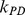 will, on the other hand, force *C* to decay in a finite time, which we call the lifetime of the charge separated state. To study this decay and determine the population (yield) of state *C*, it is convenient to solve for the evolution of 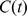 analytically. The solution is presented in the next section.

## Results

### Analytical solution of the PCT

Our goal in this section is two-fold: to obtain the conditions under which a charge separated state can be maintained in a quasi-steadystate (QSS) for a desired length of time, determined, for example, by the optimal throughput rate, and to derive simple explicit formulas for the lifetime of the QSS and the maximum yield of *C*. To this end, we first analytically solve Equation 1 for 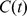. For the initial conditions, we note that the equations being homogeneous, the solutions scale with the initial population 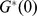, which is determined by the efficiency of the photoexcitation process. Hence, given a non-zero “source” 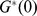 at 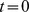, we assume that 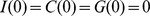, i.e., they are initially unpopulated.

We first note that Equation 1a can be integrated to give 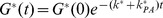. Substituting 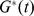 into Equation 1c for *C* and after taking a second time derivative to eliminate *I*, we arrive at the following second order equation for 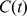:
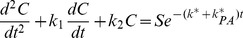
(2)where



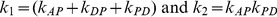
(3)The source term on the right.
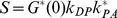
(4)


The boundary condition 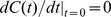 is obtained by setting 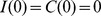 in Equation 1c. Since the coefficients appearing in Equation 2 are real, it can be written as:
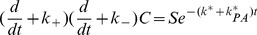
(5)where the *macroscopic* rate constants
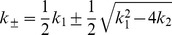
(6)are the roots of the algebraic equation: 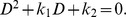 (Negative roots are used to keep the rates 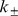 positive.) Using the following identity, where k is a constant,



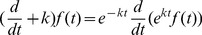
(7)we rewrite Equation 5 as
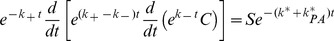
(8)


Equation 8 is now easily integrated to obtain the solution for 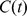. Separating the constant source term as 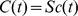, the time-dependent part 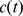 equals
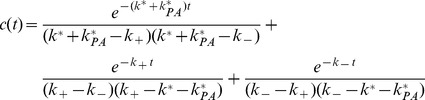
(9)This completes our derivation of 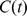.

**Figure 2:**
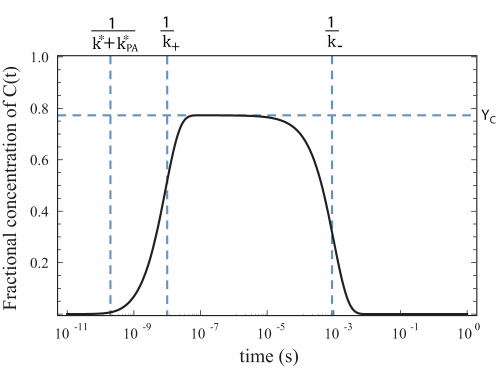
The evolution of the charge separated state 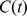 derived in eqn:ct. 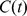
 is normalized by 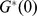, which we take to be unity. Rate constants are chosen as 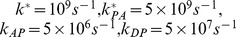, and 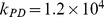 s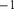. Relevant timescales are labeled on the upper axis and are marked by vertical lines (see eqn:kpm for definitions of 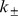). A central quasi-steadystate (QSS) plateau region is formed when these timescales are well separated. We define the decay time of the QSS, 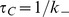, as the lifetime of the charge separated state. The horizontal line marks the yield, 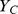, defined as the value of *C* in QSS. Analytical expressions for 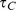 and 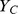 are derived in Equations 14 and 17, respectively.

A typical time evolution of 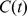 is depicted in Figure 2. This graph exhibits several aspects characteristic of charge separation in a PCT [12]: an exponential buildup of state *C* with a rate constant 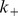, followed by a QSS plateau region that decays exponentially at the rate 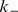. The length of time for which the QSS persists is termed the charge separation lifetime (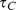), and the population of *C* at the plateau stage is termed the yield of charge separation (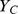). The rate 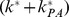 is the rate at which the state *I* is initially populated by 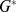 (see Equation 1b); *I* then subsequently populates *C* at the rate 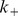. Physically, any system designed to spatially separate charges has to be able to transfer an electron from the photoexcited primary-donor 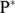 to the acceptor A (which is controlled by the rate 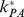) before it decays back to the ground state at the rate 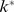. We thus restrict our analysis to
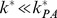
(10)


[We use the strong inequality (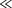) to emphasize at least an order-of-magnitude smallness.]

### The determinants of QSS lifetime and yield

In the previous section, we observed that a QSS is reached at intermediate times provided
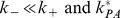
(11)


When 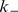 is significantly different from 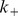 and 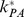, the lifetime of the QSS, and thus that of the charge separated state, can be defined as 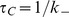. Our key observation is that 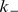 in Equation 6 can be made as small as we require by arranging either or both 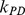 and 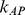 to be sufficiently small. More precisely, we find that the constraints on the macroscopic rate constants in Equation 11 are satisfied if the microscopic rates obey:
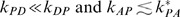
(12)


We recognize that 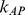 is a downhill transfer that can be fast or slow depending on the driving force of the ET determined by where it lies in the Marcus curve. 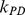, on the other hand, involves an energetically uphill electron transfer which is always slower than its corresponding downhill transfer (i.e., 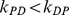). We therefore only demand a strong constraint for 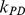 compared to that for 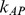 in Equation 12.

To prove that the conditions in Equation 12 are sufficient to establish a QSS, we first show that *independent* of the magnitude of 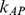 the term under the square-root in eqn:kpm, besides being positive, satisfies the stronger constraint
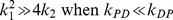
(13)


We show this by expanding the square-root in eqn:kpm and analyzing the behavior of 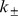 for small and large 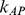. For small 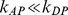, we see that 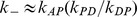 and 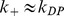, while for large 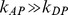, they reduce to 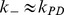 and 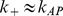. It is immediately clear that assuming 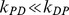 is sufficient to satisfy Equation 13 for all values of 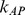. Note that, since 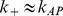 for large 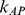, the second condition in Equation 11, namely 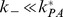, is automatically satisfied if we restrict 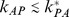. Hence, the conditions on the macroscopic rate constants in Equation 11 for a QSS to exist are met when the microscopic rate constants obey the constraints in Equation 12.

The importance of the observation that Equation 13 is satisfied for all values of 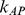 is that it allows us to expand the square-root in Equation 6 to derive simple closed-form expressions for 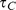 and 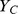. They can be analyzed to identify the key optimization parameters controlling the lifetime and yield of the charge separated state. Thus an almost exact expression for the lifetime 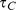 is obtained after expanding the square-root for the leading non-zero value of 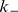


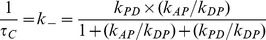
(14)


Similarly, to find the yield 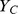, we first note in Equation 9 that the QSS behavior of 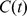 for times 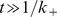 and 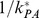 is well approximated by the surviving third term denoted below as 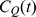.
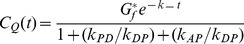
(15)

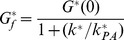
(16)


To obtain the above expressions we used Equation 11 to justify keeping only the leading order terms in the expansion of the square-root in Equation 6, namely, 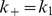 and 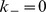. 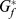 denotes the fraction of the initial population of the photoexcited state 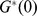 that remains after direct transition to the grounstate (predominantly fluorescence). Since 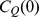 is the maximum value that 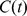 attains, namely, its value at the plateau (see Figure 2), before decaying to the groundstate, we define the yield, 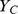, as:
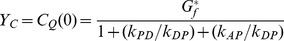
(17)


The expressions for 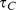 and 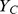 derived in Equations 14 and 17 are the main results of this section. They are compared in Figure 2 with the exact solution for 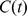 (Equation 9); the agreement is excellent. When combined with the conditions in Equation 12 for a QSS to exist, they provide all the necessary information for the design of highly optimized PCTs.

### Maximizing the QSS yield and lifetime: microscopic constraints

The advantage of having formulas Equations 14 and 17 for 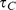 and 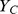 is that they enable us to identify the primary control parameters that have the largest affect on the performance of the PCT. From Equations 10–17 we conclude that the relevant ratios of the five microscopic ET rates 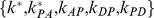 are
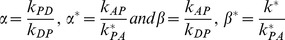
(18)


They control the formation, yield and lifetime of the charge separated state. To make the dependence explicit, we rewrite Equations 14 and 17 as functions of the dimensionless ratios here:
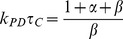
(19a)

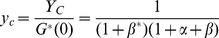
(19b)where 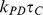 and 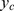 are the normalized lifetime and yield, respectively. [Note that all the individual rate constants can be expressed in terms of an appropriate combination of the dimensionless ratios and 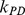.]

In terms of these ratios, the conditions for the formation of a QSS in Equation 12 translates to
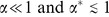
(20)


Although no fundamental restrictions on 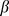 and 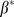 exist, it follows from Equation 19b that the yield is substantially suppressed when they are 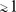. Hence, to maximize the yield, we demand
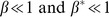
(21)


The condition 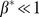 justifies the arguments leading to Equation 10 and therefore no new condition is obtained. Note that since 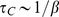, a 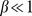 also implies long life-times. We wish to emphasize that while restricting 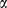 and 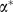 to 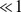 is necessary for a QSS to form, the conditions on 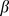 and 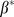 ensure a high QSS concentration or yield and a long lifetime.

This completes our analysis of the fundamental constraints on the microscopic rate constants derived to maximize the yield and the lifetime of charge separated states in the QSS regime. It is model-free in the sense that we have not utilized any particular equation to calculate the ET rates and we have not determined any specifics in terms of spatial constraints or electron affinities. We have only derived the limits of optimal values for the rate constants themselves. We now discuss in detail the physical constraints that Equations 20 and 21 impose on the energetics and architecture of the cofactor triads.

### Engineering guidelines for optimal PCTs

**Figure 3:**
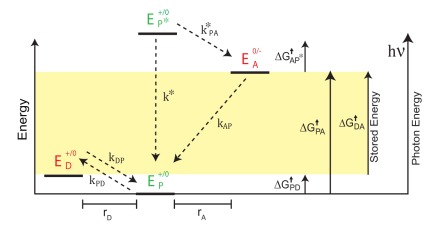
The physical characteristics of a PCT corresponding to the scheme in Figure 1C shown with the distances and reduction potentials marked explicitly. The edge-to-edge separations of the D-P pair and the P-A pair are labeled as 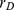 and 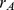, respectively. The vertical axis is in the direction of increasing energy. The respective reduction potentials are defined in terms of the half-cell potentials, 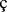 and 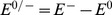 (final minus the initial state). The driving force, 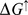, for an uphill electron transfer, say, D^+^P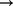 DP^+^, is defined as 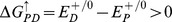. The corresponding driving force for the downhill transfer 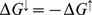. Given the driving forces and the distances, the rate constants are derived using the Moser-Dutton ruler. Note that the rate 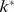, which we take to be the combined relaxation rate, fluorescent and otherwise, of the photoexcited state 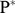 to its groundstate P, does not follow the Moser-Dutton ruler and must therefore be given. An incoming photon with the correct frequency 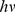 is absorbed by P to create the photoexcited state 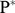. We assume that the ET rates involving 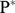
can be expressed in terms of the reduction potential of the state P, i.e., 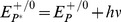, where 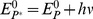 and 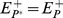. Hence the input energy 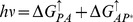. The output energy is the stored energy 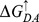 in the charge separated state.

The physical characteristics of a PCT involve the differences in reduction potentials and distances between the cofactors. Such a construct consistent with the scheme in Figure 1C is shown in Figure 3 where the energies and the distances are marked explicitly. Two more relevant metrics, geometric (overall size) and energetic (efficiency), are introduced below.

#### Separation distance

We believe that a linear construct is better because it maximizes the distance between the acceptor and donor, thus preventing relaxation by short circuiting direct electron transfer between these sites. We note, however, that this ideal is not found in all natural systems. The maximum distance of charge separation is thus:
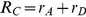
(22)where 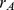 and 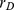 are the edge-to-edge separations of the P-A pair and D-P pair, respectively. Since the ET rates are determined primarily by the edge-to-edge distance [29], the width of P does not play a part in any of the microscopic rate constants delineated in our scheme in Figs. 1B and 3. We do not therefore include the actual width of the primary-donor site P, which even further helps to eliminate the short circuiting A
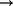
D electron transfer. Instead, we introduce a second distance parameter:
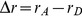
(23)that can be varied, keeping 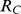 fixed, to optimize the output.

#### Charge-separation efficiency

An optimal light-activated charge separation construct should also maximize the available useful energy stored in the charge separated state 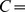 D^+^PA^−^ (see Figure 3). The energy stored in *C* can be expressed in terms of the driving force as 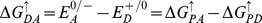 (see Figure 3). Defining the charge separation efficiency, 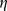, as the ratio of the stored energy 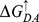 to the input photon energy, 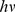, we get:
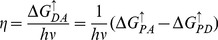
(24)


Thus, consideration of the reduction potentials of each cofactor in the PCT adds a third performance metric 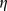, to 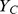 and 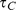, to optimize.

#### The Moser-Dutton ruler

In Equations 20 and 21 we identified and derived constraints on the ratios of the ET rate constants 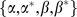 for optimal charge transfer in a PCT construct. These rates are determined by the individual values of the reduction potentials and the spatial separations of the cofactors. For this we need explicit equations that relate the rate constants to these variables. To this end, we use the Moser-Dutton ruler, a set of empirical equations that is widely used to simply and accurately predict ET rates in proteins [19], [29], [31]. The ruler predicts a rate constant, 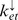, for a downhill electron transfer at room temperature, i.e., when the driving force 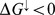 as
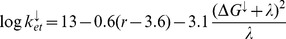
(25)

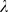
 here is the reorganization energy in eV and the term in which it appears is the Marcus term which depicts the hyperbolic dependence of the ET rate on the driving force for electron transfer [23]. Reverse or uphill electron transfer is modified by a Boltzmann term to give:



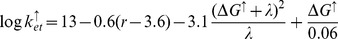
(26)The transfer rates are predicted in units of 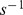. The energies 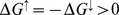 are measured in eV, and 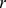 is the edge-to-edge distance between the cofactors in Å. All logs are to base 10. We note that the use of the Moser-Dutton ruler restricts our analysis from here on only to protein-based PCTs. Other PCTs can be analyzed in a similar way provided the appropriate expressions for the ET rates are used.

Using the Moser-Dutton ruler to express the microscopic variables 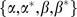 in terms of the physical variables 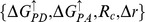 of the PCT, we get:
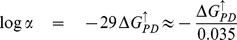
(27a)

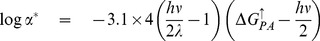
(27b)

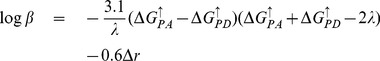
(27c)

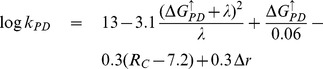
(27d)

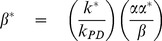
(27e)


(Refer to Equations 22 and 23 for the definitions of 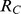 and 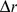 and 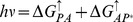.) Since 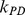 is the only dimensionful quantity we need, its form is given explicitly in Equation 27d. The last parameter 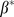 depends on 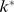, which because it involves the combined relaxation rate, fluorescent and otherwise, of the photoexcited state 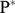 to its groundstate P, it cannot be estimated using the Moser-Dutton ruler. It is assumed to be a given quantity in our analysis. And finally, we have assumed that the reorganization energy 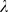 is the same for the entire construct. Only minor modifications to Equation 27c are necessary if this last assumption is relaxed. The qualitative features of our conclusions are robust, although certain quantitative predictions will have to be reworked.

Our final goal is to use the relations in Equation 27 to set general bounds on the physical makeup of a generic protein-based PCT to optimize its performance. We do this by adjusting the physical parameters 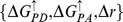 (for a fixed size 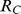 of the construct) to satisfy the constraints in Equations 20 and 21 on the rates. This way, we are guaranteed that the performance metrics 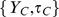 are optimized. The efficiency metric 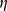 (defined in Equation 24) is then determined for a given 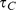 and 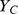. Clearly, configurations with a large difference in 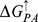 and 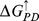 will ensure a high efficiency. It is therefore desirable to arrive at an independent set of constraints for the energies and the distances. We argue that this is mostly possible, primarily because the 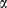 variables depend only on the driving forces and not on the separation distances (see Equations 27a and 27b). Hence any condition on the 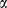 variables translates to conditions on 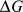, independently of the distance. These considerations are analyzed in detail next.

#### Maximizing the QSS yield and lifetime: physical constraints

In Equations 20 and 21 we identified two sets of conditions necessary for the creation of a QSS with a high charge separation yield and lifetime. We can now see what effects these constraints impose on the physical makeup of a PCT. In particular, our aim is to arrive at a set of independent constraints for the energies and distances.

#### Energy constraints - fundamental limits on the absorption wavelength of the primary-donor

From Equation 27a we note that the ratio 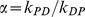 is a factor 10 smaller for approximately every 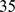 meV difference in the reduction potentials 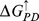. We conclude that the first constraint for the existence of a QSS, 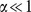 stated in Equation 20, is satisfied provided:
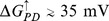
(28)


Since 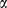 depends exponentially on 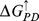, the strong inequality on 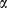 translates to a weak inequality on 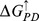. This simple observation is the key to the viability of our general mathematical analysis of a PCT. Small adjustments to the physical parameters can drive the system into the QSS regime and affect large changes in the performance metrics thus allowing us to arrive at a set of practically realizable bounds on the physical parameters.

From Equation 27b we see that the second condition in Equation 20, 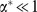, is satisfied if 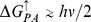 for photon energies
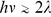
(29)


The low energy range 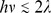 results in a small 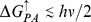 which reduces the efficiency as seen from Equation24, and is therefore not a useful range for our purpose.

**Figure 4:**
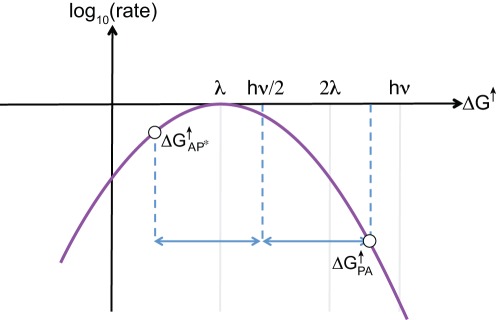
The optimal range for 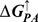 and 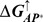 are shown on the Marcus curve. A high yield, high efficiency QSS formation in a triad requires that back electron transfer from A to P be so downhill as to be well into the Marcus inverted region. To see this, we substitute 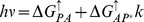 in Equation 27b so that 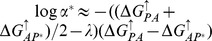, from which it immediately follows that the condition 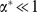 is satisfied if the mean value 
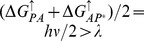
. The condition 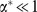 was derived in Equation 20 to be a necessary condition for the formation of a QSS.

Equation 21 presents the constraints necessary to maximize the yield 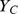. The energy dependent term for 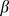 in Equation 27c is formed out of the product of the sum and difference of the driving forces. The difference term is proportional to the efficiency 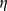 and is therefore always positive. To maintain 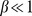 for all 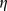, the sum must satisfy 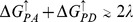. Since a large 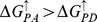 increases the efficiency from Equation 24, for practical purposes it is sufficient to ensure that 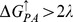. The two conditions for 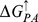 can be combined as:
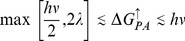
(30)where max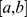 implies the larger of the two variables *a* and *b*. Thus, a 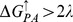 satisfies conditions for both high 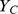 and 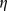, and a 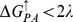 will either result in a loss of efficiency or yield. We thus predict that high yield, high efficiency QSS formation in a triad requires that back electron transfer from A to P be so downhill as to be well into the Marcus inverted region (see Figure 4.) This greatly slows the rate of this ET, allowing the donor molecule time to re-reduce the primary-donor molecule.

The upper limit is necessary to facilitate the electron transfer from the photoexcited primary-donor 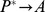. This, however, has certain limitations: as 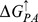 approaches the photoexcitation energy 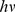, the back ET governed by the uphill rate 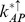 (which is set to zero in our scheme) will become relevant - see Figure 3. This will provide yet another route for the charge separated state to decay to the groundstate thus reducing the performance. Hence, we put an upper cut-off on 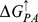. To obtain this upper cut-off, we study the behavior of the ratio of the uphill vs downhill rates using the Moser-Dutton ruler and find that (similar to the ratio 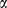 in Equation 27a)
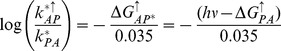
(31)


This clearly suggests that as long as the uphill driving force 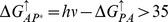 meV, the back reaction is exponentially suppressed. By direct simulation (not shown here), we find that our results obtained by setting 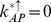 are unaffected if a difference of the order of 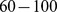 meV or higher is maintained. Although larger values will reduce the efficiency, a difference of 100 meV affects the efficiency by less than 10%.

Finally, we analyze 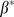 in Equation 27e. Since it can be expressed in terms of the ratios 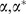 and 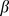, no new energy constraints can be obtained. Instead, we show below that requiring 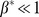 (Equation 21) provides useful insight into the geometrical construction of the PCT.

#### Distance constraints - optimal placement of the three cofactors

Since 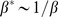 (see Equation 27e), maintaining 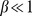 can only be done at the expense of increasing 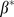 which is counterproductive as we require both 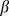 and 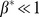 for maximum yield (Equation 21). An optimal compromise can be reached by adjusting the distance 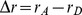. To see this, we write the 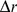 dependence explicitly in Equation 27e by combining it with Equations 33 and 34 to give 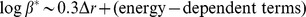. Hence, once the energy terms are optimized for a fixed total length 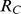, we predict that arranging for
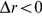
(32)will significantly reduce 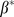. Provided 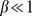, an order-of-magnitude suppression of 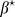 is achieved if 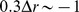, i.e., 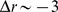 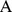. Hence, 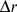 can be fine-tuned to increase the yield.

There is a relatively simple physical explanation for our prediction. This is due in part to the fact that while electron transfer rates in proteins are strongly distance dependent, the equilibria are not. As noted in Equation 19b, there are three pairs of rate constants which determine quantum yield, of which the 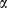 parameter involves electron transfers that are the forward and reverse of each other between P and D. Since ratios involving forward and backward rates for any particular triad are not dependent on distance, the 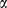 parameter is distance independent. On the other hand, the remaining two pairs, 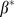 and 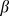, are distance dependent: the first is the initial ET between A and P which competes with the relaxation processes encompassed by 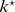. In this case the actual rate, and therefore the distance, plays a direct role in the final yield. This constrains the primary-donor and acceptor cofactors to be close enough to out-compete 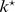. The second is the competing pair of electron transfers which can occur from state *I*: reverse electron transfer from A to P forming *G* vs. the formation of *C* by electron transfer from D to P. This second pair is a weaker constraint given the fact that electron transfer from A to P is well into the Marcus inverted region. Viewed in this light it is not surprising that optimal arrangements move the primary-donor and acceptor closer together (see Figure 4 for a demonstration of this using commonly observed parameters). The full dependence of  and 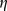 on 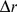 are discussed further in the discussion.

Finally, regarding the total size 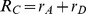: the 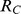 dependence of 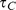 and 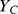 in Equations 19a and 19b are fully governed by the rate constant 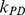 defined in Equation 27d. It follows that while the lifetime grows with 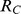 as 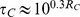, the yield is suppressed as 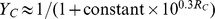.

This completes our analysis of the fundamental constraints on the physical parameters for an optimized PCT. The guidelines listed in Equations 28–32 are relevant to any protein based PCTs where the Moser-Dutton ruler is applicable.

## Discussion

In Equations 28–32, we arrived at a set of constraints on the physical makeup of a high performance PCT capable of creating and maintaining a high yield charge separated state in a QSS for a significant length of time. We now apply these results to study the efficiency of such PCTs.

While analysis of many PCT constructs focus on the charge separation lifetime, 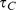 [6], it is clear that the arrangement which gives the longest possible lifetime will oftentimes make a less efficient solar energy conversion component. The charge separated state must only last as long as the mechanism for extracting this potential energy requires. After this condition is met, factors which maximize the yield and efficiency of QSS formation (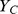 and 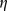) are paramount, as these determine the eventual power output. Thus, in the following we fix the lifetime 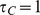 ms in our analysis.

**Figure 5:**
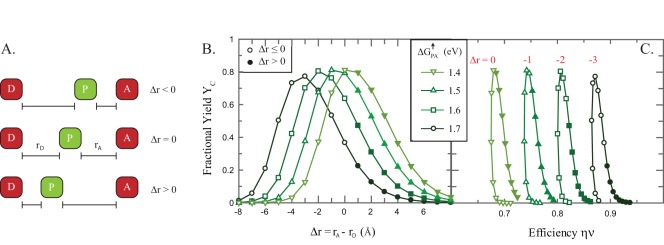
Sensitivity of the yield and efficiency of a typical PCT to 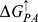 and 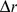. The following parameters are fixed: The light frequency 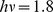 eV, the reorganization energy 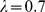 eV, the size 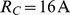, and the relaxation rate at 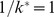 ns. The driving force 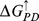 for each choice of 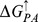 and 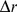 is obtained by solving Equation 19a setting 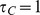 ms. All the relations necessary to invert Equation 19a for 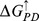 can be found in Equation 27. (A) Illustration of possible changes in 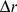 made while keeping 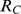 fixed. (B) Predictions for 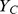 made using differing values of 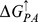 plotted as a function of 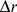. Open symbols are used to indicate 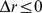 and solid symbols for 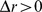. Note that in each case, a maximum 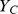 of 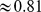 is achieved at some optimal 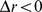. (C) Re-plot of the same data explicitly showing the variation in yield and efficiency as 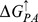 and 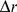 are varied. 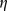 is defined in Equation 24. Legends mark different 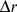 values varied in 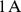 increments evaluated at the same points as in (B). The 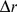 value at the maximum are labeled explicitly.

In Figure 5, we start with a set of parameters that are typical of photonic energy transduction in proteins: The reorganization energy 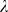 varies in the range of 0.7 to 1.4 eV for cofactors bound within typical native proteins, with 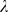 taking higher values with decreasing hydrophobicity in the local cofactor environment [19]. Light frequencies are in the near infrared and higher. We use 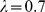 eV and 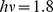 eV (690 nm) as a starting point. Note that 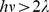 is satisfied consistent with Equation 29. To satisfy the energy constraint in Equation 30, we choose the range 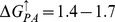 eV. [Note that the highest value for 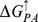 is 100 meV less than 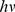 for the reasons described following Equation 31.] Electron tunneling distances in biology range from 4–14 Å, with the shorter limit that of Van der Waals contact and the longer setting a millisecond time limit on electron transfer rates [29], [31]. Hence, the sum of the distances between the cofactors are typically in the range 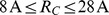; we use 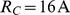. Instead of specifying the final parameter, 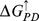, we specify the QSS lifetime 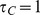 ms and solve Equation 19a for 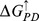 for different values of 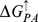 and 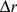 (for a fixed 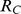). For self-consistency, we check that the 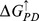 values obtained using these parameters all satisfy Equation 28 in the optimal range.

Several things are immediately apparent upon inspection of the data in Figure 5. First, the yield 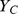 in Figure 5B is strongly dependent on 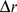, with a maximum value in each case being reached at a configuration where the primary-donor P is closer to the acceptor site than the donor site as predicted in Equation 32. Second, the efficiency 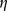 in Figure 5C is considerably enhanced as 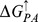 is increased closer to the maximum value 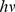.

**Figure 6:**
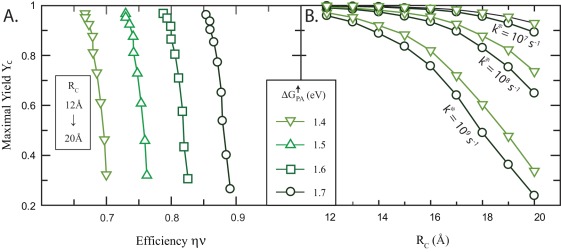
(A) Orthogonality of the yield, 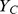, and the energy storage efficiency, 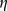, of QSS formation by the PCT. For each point, 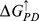 and 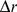 are set to the values that maximizes 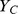 within the limits set by 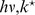 and 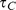 as in Figure 5. 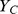 is strongly sensitive to the separation distance, 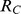, and 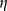 is primarily sensitive to 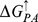. (B) The decrease in the maximal values of 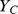 with increasing 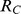 plotted for different values of 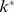 and 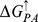. At large values of 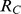 the optimized yield is primarily dependent on 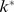.

To gain further insight on the dependence of the metrics 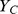 and 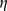 on the size 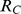 and self-relaxation (fluorescence) rate 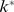, we study the variation of the optimized PCT metrics, i.e., the metrics obtained after adjusting 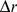 for maximum yield (i.e, we track the location of maximum yield in Figue 5 as 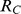 and 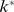 are varied). We find that once optimized for 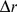, the two metrics 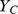 and 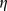 are mostly orthogonal in terms of their determinants. This is demonstrated by Figure 6A where the maximum yield is seen to be strongly suppressed with increasing 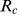 while the efficiency at maximum yield is robust. The former is due to the decrease in 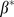 caused by the increase in distance, resulting in a smaller partitioning factor 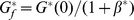 (Equation 16). As Figure 6B demonstrates, this loss can be alleviated by decreasing the rate of self-relaxation, 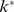, of the excited primary-donor 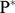.

### Conclusions

We have generated an analytical solution for the time behavior of the PCT and explored its dependence on the architecture, the reduction potentials of its components, and the absorption frequency of the primary-donor cofactor. Our primary findings are two: First, that a high efficiency, high yield PCT will have an absorption frequency more than twice the reorganization energy of the first electron transfer, and second, that the distance metric 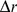 (the relative distance of the acceptor and the donor from the primary-donor) plays an important role in the determination of the yields.

We remark that our use of the Moser-Dutton ruler clearly does not capture all the subtle details of protein ET reactions. For example, the assumption that ET rates drop-off exponentially with distance ignores possible effects of the intervening medium when present [32]. Secondly, some experimental results point to an asymmetric Marcus curve [33], that is known to be relevant when certain high-frequency intramolecular vibrations are active, are not accounted for. It is a simple matter to include these effects into Equations 20 and 21, which as we noted earlier are model-free, they provide fundamental constraints on the microscopic rate constants derived to maximize the yield and the lifetime of the charge separated states in the QSS regime. Further work is needed to study the quantitative effects these corrections will have on our conclusions. We show below, however, that our analysis incorporating the simple Moser-Dutton ruler is able to successfully explain a number of remarkable features observed in Nature.

### Implications for natural systems

The first implication sets a long-wavelength limit or red-edge [34] for efficient solar energy conversion. It is estimated that the reorganization energies scale from 0.7 eV to 1.4 eV for typical proteins and cofactors bound in local environments varying from less to more hydrophobic [19]. These values predict that the longest effective wavelength for solar energy conversion is about 890 nm, correlating to the lower value. Longer wavelengths are possible, but this would necessitate a loss in either yield or energy. Our analysis is primarily limited by the fact that we include only three discrete sites for electron localization. Natural photosynthetic proteins have additional acceptor molecules, which enable the stepwise diffusion of the electron further away from the primary-donor. Their effect on the behavior of the PCT is unclear.

**Figure 7:**
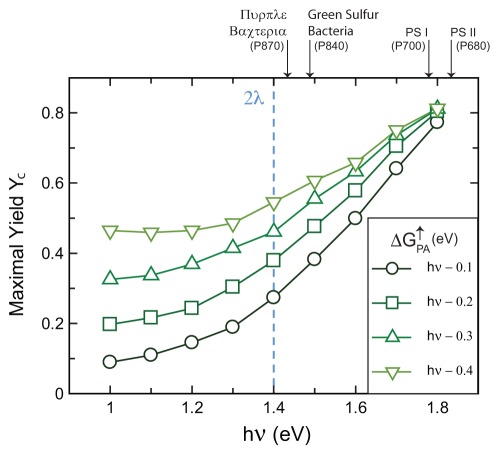
Predicted long-wavelength limit or red-edge for efficient solar energy conversion. Photon energies smaller than 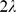 cause a loss in either yield or energy storage efficiciency. For each point, the value of 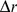 used maximizes 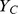 within the constraints set by 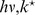 and 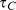 as in Figure 5. The 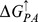 values are calculated as 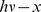 where 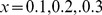 and 0.4 eV. Wavelength limits of natural systems depicted above the axis are taken from [34].

However, we do note in Figure 7 that at present the observed wavelength limits for oxygenic photosynthesis, an energetically demanding process in that it must create oxidizing potentials high enough to oxidize water [2], are within the values [35] we predict. Furthermore the limits observed for charge separation in any natural organism, those from bacterial photosynthesis [34], are within 150 nm, or 160 meV, of that predicted by our model, as shown in Figure 7. This suggests that efforts to re-engineer natural systems to utilize longer wavelengths of light, and thus garner a greater fraction of the solar emission spectrum [36] will result in considerable losses of either yield or conversion efficiency to do so.

**Figure 8:**
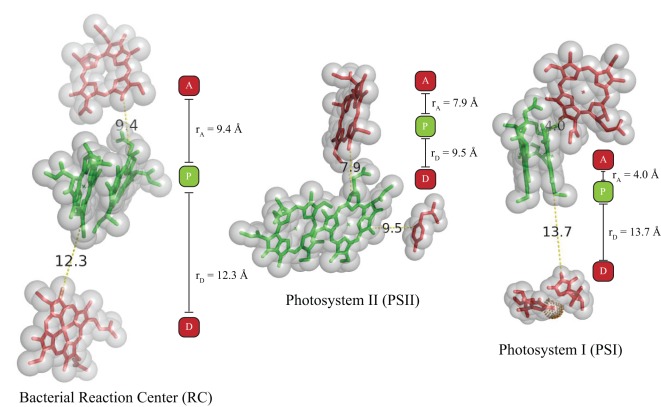
Representative structures of natural photosynthetic cofactor triads. Primary donors P are colored green with the donor D and acceptor A cofactors colored red in each structure. Distances are measured edge-to-edge. (left) Reaction Center complex from *Blastochloris viridis* (PDB ID 2X5U) [37], (center) Photosystem II from *Thermosynechococcus elongates* (PDB ID 3BZ1) [38], and (right) is Photosystem I Plastcyanin complex from *Prochlorothrix hollandica* created by computational docking [39]. Images and distances were created using Pymol.

The other prediction is that yields are maximized by placing the primary-donor closer to the acceptor than the donor cofactor. This again may be altered when further discrete electron binding sites are added to the construct, but we again note that for the limited subset of photosynthetic proteins which have structures which include the donor cofactor, the primary-donors are indeed positioned in this manner (Figure 8).

### Engineering parameters for artificial charge separation constructs

This analysis sets out the optimal physical composition of an artificial protein-based charge separation construct. It demonstrates that efficient, high yield charge separation can be engineered with 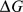 values that are both feasible to engineer and within the ranges observed in natural systems. It further identifies the molecular properties which are important targets for engineering improved PCTs. Principle among these is the control of the reorganization energy, 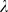. A smaller value of 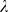 will enable the utilization of longer wavelengths of light, enabling the possible utilization of a larger fraction of the solar emission spectrum. There have been very few experimental determinations of 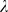 values within a protein, and even less work on manipulating or optimizing its magnitude. However, it is apparent that it will be important to be able to manipulate this parameter effectively.

While we have identified 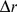 as a critical parameter for high yield constructs, at smaller cofactor separation distances the tolerances for 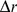 are very small. The large changes engendered by even a 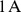 change in 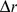 make high yield small constructs difficult to create. Larger constructs have broader 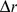 maxima, but in this case yields are reduced due to unproductive primary-donor relaxation rates, or 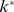 (see Equation 16). Consequently the creation of primary-donor cofactors with longer excited state lifetimes is paramount. As Figure 6B demonstrates, longer lifetime cofactors will enable significantly larger constructs, and thus eases the optimization of 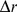. We further note that while our analysis uses the protein-specific Moser-Dutton ruler, which models coupling as an exponential drop-off in electron transfer rate with distance, the model-free portion of this analysis leading to Equations 20 and 21 are applicable to synthetic constructs as well. The distance dependence in these systems depends strongly on the nature of the bridging elements which connect the triad cofactors, and the analysis presented here predicts that the coupling must in general be as strong as possible between the primary-donor and acceptor. In a protein this means putting them close together since the “bridge” is always the same. In a bridged system this means choosing a bridge that maximizes the coupling, but it doesn’t necessarily mean bringing them closer together.
