# Correction: Fundamental Limits on Wavelength, Efficiency and Yield of the Charge Separation Triad

**DOI:** 10.1371/annotation/7db1b9ef-c93c-4b02-b721-a8473d2bb4e2

**Published:** 2012-08-09

**Authors:** Alexander Punnoose, Liza A. McConnell, Wei Liu, Andrew C. Mutter, Ronald L. Koder

The second and fifth authors' names were spelled incorrectly. The correct name for the second author is Liza A. McConnell, and the correct name for the fifth author is Ronald L. Koder. The correct citation is: Punnoose A, McConnell LA, Liu W, Mutter AC, Koder RL (2012) Fundamental Limits on Wavelength, Efficiency and Yield of the Charge Separation Triad. PLoS ONE 7(6): e36065. doi:10.1371/journal.pone.0036065 

